# A Retrospective Clinical Analysis of the Serum Bile Acid Alteration Caused by Traumatic Brain Injury

**DOI:** 10.3389/fneur.2021.624378

**Published:** 2021-08-25

**Authors:** Yuanrun Zhu, Zijian Chen, Wendong You, Yadong Wang, Mengdi Tu, Peidong Zheng, Liang Wen, Xiaofeng Yang

**Affiliations:** ^1^The First Affiliated Hospital, School of Medicine, Zhejiang University, Hangzhou, China; ^2^Zhejiang University School of Medicine, Hangzhou, China; ^3^Shaoxing Hospital of Traditional Chinese Medicine, Zhejiang Chinese Medical University, Shaoxing, China

**Keywords:** traumatic brain injury, bile acid, retrospective study, brain-gut axis, gut microbiota

## Abstract

Traumatic brain injury (TBI) can cause damage to peripheral organ systems, such as digestive organ system, and alterations of gut microbiota in addition to brain injury. Our previous study found that TBI induced gastrointestinal dysfunction accompanied by alterations of bile acid metabolism. Bile acid and its receptors have been reported to play important roles in various neurological diseases. To further examine the changes of bile acid metabolism in TBI patients, we performed a retrospective clinical analysis. In this study, 177 patients were included, and the results showed that TBI patients had more frequent antibiotic use compared with a control group. Regression analysis identified TBI as an independent factor for reduction of serum bile acid level (B = −1.762, *p* = 0.006), even with antibiotic use taken into a regression model. Sub-group regression analysis of TBI patients showed that antibiotic use was negatively associated with bile acid level, while creatinine and triglyceride were positively associated with bile acid level. In conclusion, these data indicated that TBI could greatly reduce serum bile acid. This study provided preliminary but novel clinical evidence of TBI interfering with bile acid metabolism, and further studies with large sample sizes are needed to validate these findings in the future.

## Introduction

Traumatic brain injury (TBI) is a worldwide major cause of death and disability and leads to heavy medical burden to the society (1). TBI not only deals primary damage to the central nervous system (CNS) but also causes secondary disorder to multiple peripheral organs (2–4). One of the most affected peripheral systems is the digestive system, which will suffer various pathological changes and present with different kinds of abnormalities (5, 6). These abnormalities include digestive dysfunction, gut inflammation, barrier damage, gut microbiota disturbance, and other gastrointestinal changes. Clinical expressions caused by these changes are also common. More than 50% of TBI patients suffer from gastrointestinal symptoms, and the rate of severe complications such as gastrointestinal hemorrhage varies from 5 to 12% in different studies (7–9). This high incidence of gastrointestinal complications has drawn the attention of researchers to relevant physiological or pathological progresses.

Bile acid metabolism is an important physiology progress which involves the intestine, gut microbiota, liver, and gallbladder (10, 11). Bile acid is related to multiple diseases and has been considered to be a potential therapy tool especially in gastrointestinal abnormalities (12). Apart from its close connection to the digestive system, it also showed influence on neurological diseases especially in stroke (13) and intracranial hemorrhage (ICH) (14). Bile acid possibly plays a positive role in stroke through the activation of its receptors such as TGR5 and is able to attenuate neuroinflammation through different pathways, including neuron apoptosis inhibition and microglia functioning (15, 16). Moreover, bile acid reduction has been reported to correlate with higher stroke risk in a prospective study (17). In recent studies, bile acid and its receptors have also been reported to connect with TBI and related pathological changes (18–21). Experimental evidence indicates that bile acid transporters expressed by hypothalamus neurons are changed post injury. Certain bile acids and analogs are also able to influence the neuroinflammation and neuron apoptosis after TBI events. These results are promising and indicate that bile acid has a role in the disease progress of TBI.

Given the potential function of bile acid, researchers have been making efforts to figure out whether TBI could alter bile acid metabolism. The existing hypothesis that TBI and bile acid have bidirectional interaction is supported by considerable experimental evidence. Bile acid metabolism has been well-acknowledged to rely on the gut microbiota (22, 23). It was also reported in a previous study that TBI could significantly influence the intestinal bacteria and reduce its abundance (24, 25). Our recent study has proved that the microbiota change was accompanied by bile acid alteration in serum and feces. Specifically, the serum bile acid changes of mice involved both primary and secondary bile acids (26). Under such circumstance, it is natural for researchers to consider TBI affective to bile acid through gut microbiota. Yet, the current studies are mostly confined to animal models. Compared with experimental studies, direct clinical evidence showing TBI influences bile acid metabolism is still lacking.

In this study, based on existing laboratory evidence, we intended to research the influence of TBI events on the bile acid metabolism through a retrospective clinical analysis involving patients with or without TBI. We also did a sub-group analysis to study other potential factors that possibly affect bile acid level in TBI patients.

## Methods

### Patient Population

This retrospective study screened TBI and trigeminal neuralgia (TN)/hemifacial spasm (HFS) patients who were admitted to our medical center from January 2019 to January 2020. The inclusion required patients to have laboratory test results from standard fasting blood samples upon admission, which should include serum bile acid level as well. The exclusion criteria included (1) patients had original hepatobiliary, pancreatic, or gastrointestinal diseases; (2) patients had been under full parenteral nutrition support which would cause decreased bile acid synthesis (27); (3) patients were 1 week post any sort of surgical procedure or non-TBI trauma events; and (4) patients had already shown multi-organ failure or other terminal stage performance.

The TN/HFS patients were included in this study as a control group. These TN/HFS patients were not accompanied by CNS injuries, and TN/HFS had never been found related to bile acid metabolism. Since serum bile acid level is not routinely included in the periodic health examination service of our center, the data of healthy volunteers were not available in this single-center retrospective study.

### Blood Sample Details

This study set a relatively strict standard inclusion criteria regarding blood sample acquirement so as to obtain reliable bile acid data. The test results of included patients were all obtained by our medical center so that deviation between different labs could be prevented. The blood samples were obtained on the morning of the admission after at least 8-h fasting. This would be before any invasive treatment for patients, so that they were at a most consistent status. Specifically, the laboratory tests should at least cover liver function, kidney function, lipid metabolism, serum bile acid, blood routine, and coagulation function.

It is worthy to note that this strategy would not include emergency TBI patients since their pre-operational tests were urgent and would not meet the inclusion criteria. Mild TBI patients would not be included either since they did not need to be hospitalized.

### Data Collection

Collected clinical data included gender, age, diagnosis, medical history, initial Glasgow Coma Scale (GCS) upon first admission, alanine transaminase (ALT) level, aspartate aminotransferase (AST) level, antibiotic use, creatinine level, total bilirubin, cholesterol level, triglyceride level, and serum bile acid level. For TBI patients, the post-trauma duration and injury category are also documented. No personal information is traceable in this report, and the study is in keeping with the ethical standards of the 1964 Declaration of Helsinki and its later amendments. The study is approved by the ethics committee of our medical center with the approval number 2020IIT1102.

### Statistical Analysis

Statistical analysis was performed using SPSS version 19.0 (IBM Corp, Armonk, NY). Continuous variables are given as means and standard deviations. Categorical variables are given as numbers and percentages. To compare continuous variables, Student's *t*-test (if normally distributed) or Mann–Whitney *U*-test (if not normally distributed) were performed. To compare categorical variables, chi-square test was performed. Linear regression analysis was performed to determine the factors that influence serum bile acid level, and variance inflation factor (VIF) values were calculated in the process to rule out the possible impact of multi-colinearity. Statistical significance was determined when *p* < 0.05.

## Results

### Patient Inclusion and Basic Characteristics

**Figure 1:**
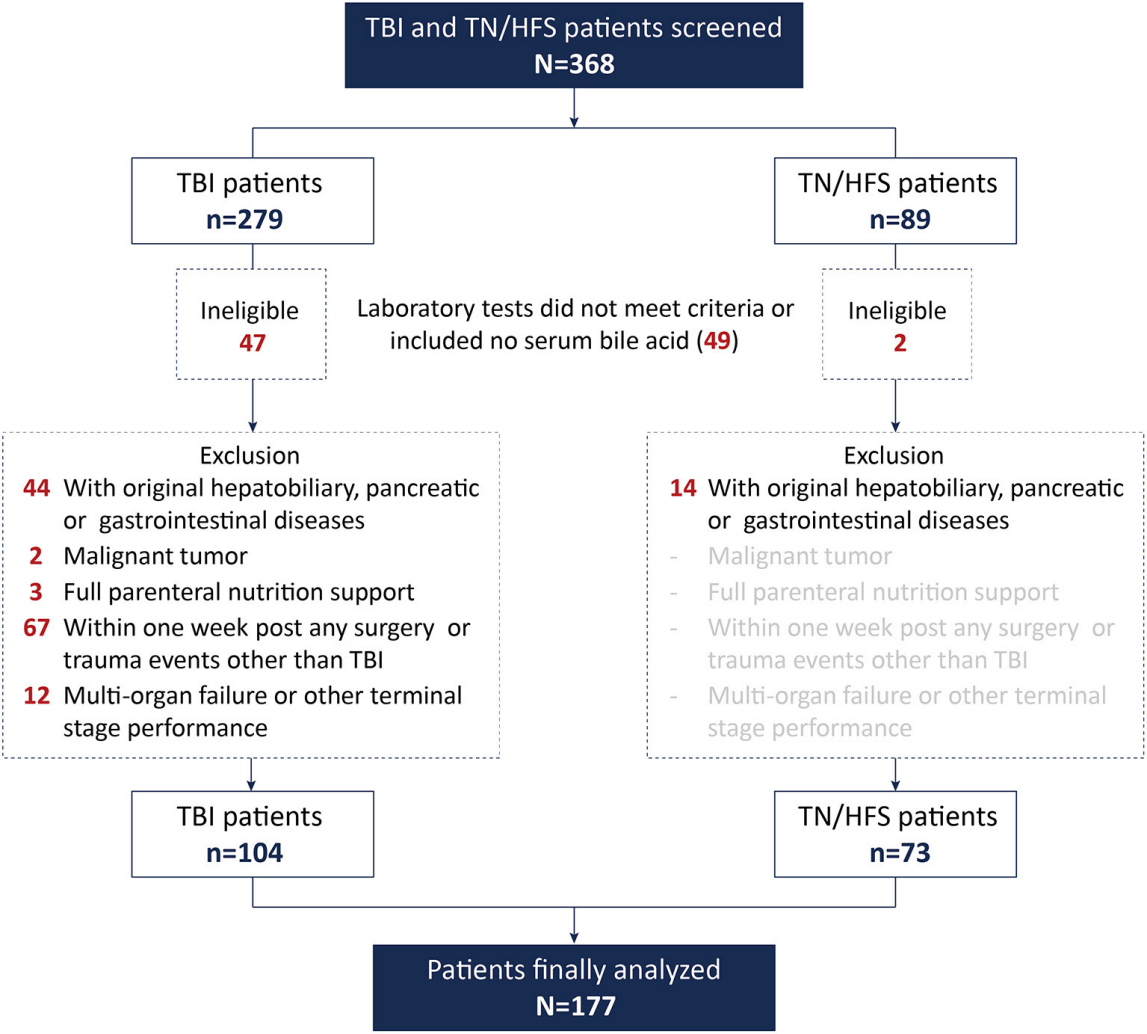
Details of patient inclusion.

We retrospectively screened 368 TBI or TN/HFS patients, among whom 177 were analyzed after exclusion. The inclusion flow chart is shown in Figure 1. It is worthy to note that no patients with repeated TBI were finally included.

Among the 177 patients included in this study, 104 (58.8%) were TBI patients. The average age of all 177 patients was 57.6 ± 13.2 years, and 103 (58.2%) patients were male. The initial GCS was 13.1 ± 3.19. Thirty-eight (21.5%) patients were under antibiotic treatment when the blood samples were obtained. The serum bile acid level was 5.18 ± 3.41 μmol/L. Twenty-one patients had a history of hypertension, seven had benign tumor, one patient had a history of chronic nephritis, four had a history of heart disease, and two had a history of asthma. TBI patients included 79 with cerebral contusion and/or laceration, 11 with only epidural hematoma, 6 with only subdural hematoma, 4 with epidural and subdural hematoma, and 4 with only skull fracture (no intracranial lesion). The detailed basic characteristics can be found in Table 1.

**Table 1:** Characteristics of patients.

**Characteristic**	**Value**
Number of patients	177
Gender, *n* (%)	
Male	103 (58.2%)
Female	74 (41.8%)
Age, mean ± SD (years)	57.6 ± 13.2
Serum bile acid, mean ± SD (μmol/L)	5.18 ± 3.41
Total bilirubin, mean ± SD (μmol/L)	9.38 ± 4.89
ALT, mean ± SD (u/L)	21.7 ± 18.5
AST, mean ± SD (u/L)	22.3 ± 13.7
Antibiotics, *n* (%)	38 (21.5%)
Creatinine, mean ± SD (μmol/L)	61.8 ± 14.5
GCS, mean ± SD	13.1 ± 3.19
Cholesterol, mean ± SD (mmol/L)	4.27 ± 0.95
Triglyceride, mean ± SD (mmol/L)	1.46 ± 1.14
Medical history (other than exclusion criteria)	
Hypertension	21
Benign tumor	7
Chronic nephritis	1
Heart disease	4
Asthma	2
TBI, *n* (%)	104 (58.8%)
With cerebral contusion and/or laceration	79
Epidural hematoma	11
Subdural hematoma	6
Epidural and subdural hematoma	4
Only skull fracture	4

*SD, standard deviation; ALT, alanine aminotransferase; AST, aspartate aminotransferase; GCS, Glasgow Coma Scale; TBI, traumatic brain injury*.

### Differences Between Patients With or Without TBI

The median post-injury time of sample collection for TBI patients was 30.0 days. Compared with the control group, TBI patients had higher ALT (24.5 ± 21.8 vs. 17.7 ± 11.3 u/L, *p* = 0.007) and AST (25.0 ± 16.8 vs. 18.5 ± 5.70 u/L, *p* < 0.001) levels, but their creatinine (59.0 ± 14.9 vs. 65.8 ± 13.0, *p* = 0.002) and cholesterol (4.08 ± 0.96 vs. 4.54 ± 0.87, *p* = 0.002) levels were lower. TBI patients also had a greater ratio of antibiotic use (36.5 vs. 0%, *p* < 0.001) and lower initial GCS (11.7 ± 3.59 vs. 15.0 ± 0.00, *p* < 0.001) because TN/HFS patients had no need of anti-infection treatment before surgery and suffered no CNS damage leading to unconsciousness. The serum bile acid level of TBI patients was about two-thirds of that in the control group patients, and the difference was statistically significant (4.31 ± 2.70 vs. 6.41 ± 3.93 μmol/L, *p* < 0.001). The age, total bilirubin, and triglyceride were similar between the two groups. The differences are presented in Table 2.

**Table 2:** Differences between patients with or without TBI.

**Characteristic**	**TBI**	**Without TBI**	***p*-value**
Number of patients	104	73	
Median post-injury time of sample collection (days)	30.0	-	
Gender (female)	30 (28.8%)	44 (60.3%)	<0.001
Age	58.0 ± 14.1	57.1 ± 11.8	0.670
Total bilirubin (μmol/L)	9.83 ± 5.10	8.73 ± 4.55	0.141
ALT (u/L)	24.5 ± 21.8	17.7 ± 11.3	0.007
AST (u/L)	25.0 ± 16.8	18.5 ± 5.70	<0.001
Antibiotics	38 (36.5%)	0 (0%)	<0.001
Creatinine (μmol/L)	59.0 ± 14.9	65.8 ± 13.0	0.002
GCS	11.7 ± 3.59	15.0 ± 0.00	<0.001
Cholesterol (mmol/L)	4.08 ± 0.96	4.54 ± 0.87	0.002
Triglyceride (mmol/L)	1.34 ± 0.80	1.62 ± 1.50	0.124
Serum bile acid (μmol/L)	4.31 ± 2.70	6.41 ± 3.93	<0.001

*ALT, alanine aminotransferase; AST, aspartate aminotransferase; GCS, Glasgow Coma Scale; TBI, traumatic brain injury*.

### The Influence of TBI on Serum Bile Acid Level

Given the different characteristics of TBI patients compared with the control group, we performed regression analysis to determine whether TBI has independent effect on bile acid metabolism. The results indicated that TBI was the only independent factor that had statistically significant influence on serum bile acid level in our study cohort (B = −1.762, 95% CI: −3.020 to −0.505, *p* = 0.006). Other factors including antibiotic use and initial GCS were not independently effective when TBI was included in the regression model. The regression analysis of all patients is shown in Table 3.

**Table 3:** Linear regression analysis for serum bile acid level of whole cohort.

**Factors**	**B**	**Standard error**	***p*-value**	**95% CI**	**VIF**
Gender	0.555	0.605	0.360	−0.640 to 1.751	1.527
Age	0.038	0.019	0.051	0.000 to 0.076	1.093
Total bilirubin	−0.102	0.053	0.055	−0.206 to 0.002	1.129
ALT	−0.009	0.014	0.529	−0.037 to 0.019	1.166
Antibiotics	−1.319	0.704	0.063	−2.709 to 0.071	1.431
Creatinine	0.012	0.021	0.568	−0.029 to 0.053	1.530
GCS	−0.038	0.101	0.709	−0.236 to 0.161	1.749
Cholesterol	−0.216	0.292	0.461	−0.793 to 0.361	1.319
Triglyceride	0.120	0.232	0.606	−0.338 to 0.577	1.200
TBI	−1.762	0.637	**0.006**	−3.020 to −0.505	1.683

*ALT, alanine aminotransferase; GCS, Glasgow Coma Scale; TBI, traumatic brain injury; VIF, variance inflation factor. Bold value indicates p < 0.05*.

It is worthy to note that the AST and ALT levels presented a co-linearity problem that cannot be ignored; thus, in the regression analysis (both in this section and the sub-group section), only ALT was included.

### The Influence of Other Factors on Serum Bile Acid Level in TBI Patients

To explore the potential effect of other factors on bile acid level in TBI patients, we also did sub-group analysis using regression method. The data showed that in the TBI group of our study, antibiotic use (B = −1.204, 95% CI: −2.356 to −0.052, *p* = 0.041) was associated with a lower serum bile acid level, while creatinine (B = 0.051, 95% CI: 0.009–0.093, *p* = 0.018) and triglyceride (B = 0.758, 95% CI: 0.032–1.485, *p* = 0.041) had positive association with the serum bile acid level. Other factors including gender, age, post-injury time, total bilirubin, ALT, GCS, and cholesterol did not independently influence serum bile acid according to our dataset. The detailed sub-group analysis is presented in Table 4.

**Table 4:** Linear regression analysis for serum bile acid level of TBI patients.

**Factors**	**B**	**Standard error**	***p*-value**	**95% CI**	**VIF**
Gender	0.245	0.665	0.714	−1.075 to 1.565	1.402
Age	−0.003	0.020	0.884	−0.042 to 0.036	1.155
Post-injury time (days)	−0.002	0.003	0.398	−0.008 to 0.003	1.117
Total bilirubin	−0.069	0.054	0.203	−0.177 to 0.038	1.163
ALT	−0.006	0.013	0.649	−0.031 to 0.019	1.174
Antibiotics	−1.204	0.580	**0.041**	−2.356 to −0.052	1.207
Creatinine	0.051	0.021	**0.018**	0.009 to 0.093	1.544
GCS	−0.092	0.086	0.286	−0.263 to 0.078	1.456
Cholesterol	−0.084	0.315	0.790	−0.711 to 0.542	1.416
Triglyceride	0.758	0.366	**0.041**	0.032 to 1.485	1.324

*ALT, alanine aminotransferase; GCS, Glasgow Coma Scale; TBI, traumatic brain injury; VIF, variance inflation factor. Bold value indicates p < 0.05*.

## Discussion

In this study, we illustrated that TBI might interfere with bile acid metabolism and, as a result, reduce the serum bile acid level. These results provide novel clinical evidence regarding the relationship between bile acid and TBI. Hopefully, they can work as a clue for future bile acid study in neuroscience.

As mentioned, the role of bile acid and its receptors in neurological diseases has been noticed by researchers a decade ago. Within the recent several years, multiple studies have reported that bile acid receptors or transporters [such as TGR5 and apical sodium-dependent bile acid (ASBT) transporter] were involved in the pathological progress after brain injury in animal models (15, 20). Bile acid receptors and transporters were reported to decrease as early as 2 h post injury, and ASBT-transporter expressing neurons in the hypothalamus area were revealed to suffer a significant decrease following TBI events (19). The activators of these receptors also showed biological effects *in vivo* (21). Such results all indicated that bile acid had potential significance in TBI pathologies. However, there has been limited clinical evidence linking TBI with bile acid, and it was unclear whether TBI could directly disturb the normal bile acid circulation in patients. Our data indicated that TBI could make a difference to bile acid metabolism. This relationship existing in patients might put more confidence in future studies focused on bile acid function in TBI treatment.

In previous studies, bile acid metabolism was reported to be influenced by gut microbiota (23). This could be one of the mechanisms by which TBI changes serum bile acid level since TBI is able to disturb intestinal bacteria (25). As mentioned, our recent results in TBI in mice proved that the microbiota change was accompanied by bile acid alteration in serum and feces, which put more reliability on the relationship. The possible mechanism behind this correlation could be related to the characteristic of bile acid metabolism. It has been widely accepted that bile acid is one of the major metabolites of gut microbiota (28). To be noted, in our previous study, both primary and secondary bile acid levels were reduced. Since primary bile acids are synthesized in the liver and then secreted into the intestinal tract, it is possible that apart from the microbiota, the influence of TBI on gastrointestinal absorption function may also be important, which needs further studies to confirm. Of course, TBI is not the only factor in microbiota alteration. Antibiotics, especially broad-spectrum categories, are highly effective in reducing gut microbiota abundance. In fact, antibiotics are well-accepted tools in building intestinal germ-free animal models (29, 30). At the same time, TBI patients often have much higher rates of antibiotic use than normal people. This could bring possible bias (31, 32). In our study, TBI patients had been given antibiotics much more frequently than control group patients. Thus, to avoid bias, we performed regression analysis. It turned out that even when taking antibiotic use into consideration, TBI still acted as an independent risk factor for serum bile acid reduction.

Apart from injury events, bile acid metabolism is also supposed to be associated with other physiological progresses (33, 34). In our regression analysis, due to the strong effect of TBI, all other factors showed no statistical significance. To gain further understanding, we also performed sub-group analysis in TBI patients. The results indicated antibiotics, serum creatinine, and triglyceride were related to the change of serum bile acid level. Antibiotics are able to further reduce bile acid level in TBI patients possibly due to their effects on microbiota as mentioned. Triglyceride showed a positive correlation with bile acid level, which might indicate the relationship between the bile acid and the lipid metabolism. It is worthy to note that in our study cholesterol is not significantly related to bile acid level, and the reason is not very clear. Serum creatinine also showed association with bile acid, but compared with triglyceride and antibiotics, its influence was rather minor according to the regression results. The correlation analysis of non-TBI patients indicated that the positive correlations of both triglyceride and creatinine were unique in TBI patients (see Supplementary Table 1). It is possible that the central nervous injury drives these related variations of different metabolism processes. Given the limitations of our study, based on current data it is hard to confirm these relationships and their clinical meanings; thus, further large-scale study is needed.

We have to state that this study had included limited amount of patients and the control group did not consist of healthy volunteers because of the retrospective design. Thus, apart from the strong effects of TBI and antibiotics, the influence of other factors such as creatinine on bile acid level might not be universal and should be interpreted with caution. Moreover, many TBI patients requiring immediate operation at the time of admission were not included because their pre-operational laboratory tests would only be minimally essential for the neurosurgical procedure. As a result, they would not meet the inclusion criteria. Mild TBI patients who did not need hospitalization were out of our study scope either, which made it difficult to determine whether the injury severity would affect bile acid level. Given these limitations, more large-scale studies will be needed to determine the biological role of bile acid metabolism in TBI disease and its interaction with other physiological progresses.

In conclusion, TBI is an independent factor that is able to lower serum bile acid level. Among TBI patients, antibiotic use will reduce bile acid further. Higher triglyceride and creatinine are possibly related to higher serum bile acid level in TBI patients, which requires more detailed studies to confirm in the future.

## Data Availability Statement

The original contributions presented in the study are included in the article/Supplementary Material, further inquiries can be directed to the corresponding authors.

## Ethics Statement

The studies involving human participants were reviewed and approved by The First Affiliated Hospital, School of Medicine, Zhejiang University. The patients/participants provided their written informed consent to participate in this study.

## Author Contributions

YZ, ZC, WY, YW, MT, PZ, LW, and XY conceived and planned the study. YZ, ZC, WY, YW, MT, and PZ carried out the data collection and analysis. LW and XY checked and supervised the collected data and analysis progress. YZ, ZC, and WY took part in writing the manuscript. All authors provided critical feedback and helped shape the research, analysis, and manuscript.

## Conflict of Interest

The authors declare that the research was conducted in the absence of any commercial or financial relationships that could be construed as a potential conflict of interest.

## Publisher's Note

All claims expressed in this article are solely those of the authors and do not necessarily represent those of their affiliated organizations, or those of the publisher, the editors and the reviewers. Any product that may be evaluated in this article, or claim that may be made by its manufacturer, is not guaranteed or endorsed by the publisher.
