# Supplementary material for: A Retrospective Clinical Analysis of the Serum Bile Acid Alteration Caused by Traumatic Brain Injury
Source: Front Neurol. 2021 Aug 25;12:624378. doi: 10.3389/fneur.2021.624378 (PMC8424180; doi:10.3389/fneur.2021.624378)
Supplement: Supplementary file 1 [file Table_1.DOCX]

**Table S1. Correlation of triglyceride/creatinine levels and bile acid levels within TN/HFS group**

|  |  | **Bile acid** | **Triglyceride** | **Creatinine** |
| --- | --- | --- | --- | --- |
| **Bile acid** | Pearson correlation | / | -0.038 | -0.030 |
|  | P value | / | 0.753 | 0.800 |
| **Triglyceride** | Pearson Correlation | -0.038 | / | -0.031 |
|  | P value | 0.753 | / | 0.796 |
| **Creatinine** | Pearson Correlation | -0.030 | -0.031 | / |
|  | P value | 0.800 | 0.796 | / |
